# Incentive Strategies for Low-Carbon Supply Chains with Asymmetric Information of Carbon Reduction Efficiency

**DOI:** 10.3390/ijerph15122736

**Published:** 2018-12-04

**Authors:** Qinpeng Wang, Longfei He

**Affiliations:** 1 School of Management Science and Engineering, Hebei University of Economics and Business, Shijiazhuang 050061, China; qinpeng@heuet.edu.cn; 2 College of Management and Economics, Tianjin University, Tianjin 300072, China

**Keywords:** low-carbon supply chain, information asymmetry, pooling equilibrium, separating equilibrium, sustainability

## Abstract

Information concerning carbon reduction efficiency is of great significance to supply chain operations. Considering the impact of information asymmetry on the performance of low-carbon supply chain, we therefore analyze a chain system with a single product designer and a single manufacturer. The manufacturer owns information on carbon reduction efficiency, whereas the product designer only knows that the carbon reduction efficiency of the manufacturer is either high or low. To induce the manufacturer to reveal his true private information of carbon-reduction efficiency to the product designer, we devise the pooling and separating equilibrium models to compare the impacts of these two models on supply chain performance, respectively. We find that the high-efficiency manufacturer gets his first-best choice at the equilibrium decision in the separating model, and obtains the information rent in the pooling model. The information rent increases in the efficiency difference between the two emission-reduction types. Additionally, we examine how the probability of the high (or low)-efficiency manufacturer being chosen impacts on both the profits of chain members and carbon-reduction levels. The research provides a reference for companies about how to cooperate with partner who possess private information of carbon emissions.

## 1. Introduction

The industrial companies become the principal parts of executing carbon reduction since the worldwide industrialization process should account for the substantial increase of carbon emission, according to Magazzino [1] which examine the causal link between Gross Domestic Product (GDP) and energy by analyzing relationship among economic growth, carbon emissions, and energy use for 19 Asia-Pacific Economic Cooperation (APEC) countries over 45 consecutive years. Accordingly, the industrial firms as well as their supply chains will be affected by the governmental regulative policies of carbon reduction. However, firms generally deal with affairs based on their acquaintance, namely, relevant information. Similarly, channel members in a supply chain located in different links make decisions to maximize their own profits also in terms of what information they have. In fact, the information asymmetry thereby widely exists in a supply chain, such as cost and demand information asymmetry. Channel members with information advantages may gain more profit by sacrificing the benefits of the information disadvantage, which may render the whole channel inefficient. In fact, information asymmetry usually exists in reality. Reduction of carbon dioxide emissions and other greenhouse gases discharged to the atmosphere is the common aim of global supply chains as part of their social responsibility (Benjaafar et al. [2], Lee [3]), which has been extensively researched in academia. Such as Du et al. [4], Du et al. [5], He et al. [6], He et al. [7], and He et al. [8], in which the authors assume that the information of carbon emission reduction efficiency is complete. In fact, the manufacturers would not like to disclose their information. Conversely, the private information possessed by firms may help them earn more profits, whereas this may lead to inefficiency for the whole channel. By avoiding the above questions, many companies have tried to enhance the visibility of carbon reduction efficiency or cost. Huawei, a worldwide leading manufacturer of information and communication equipment, requires its suppliers to disclose their carbon emissions information and dedicates itself to decreasing the carbon emissions of its whole supply chain (Huawei Sustainability Report [9]). Apple Inc. launched its supplier clean energy program in 2015 to reduce emissions from its supply chain (Liang [10]). Siemens also stipulates the same carbon reduction requirement for its suppliers, helps its customers reduce carbon emissions and even actively participates in partners’ carbon reduction process by sending its engineers to identify carbon-reduction opportunities and provide related services (Siemens sustainability information [11]). Lenovo and its contract manufacturer, Compal Electronics, use raw material preparation and other smart manufacturing methods to achieve climate change policy goals (Thomsen [12]).

Related studies (like Rodrigo et al. [13]) show that consumers’ low-carbon preference has become a significant driver of supply chains to reduce carbon emissions in the context of the Paris Agreement. To attract carbon sensitive consumers, an important factor in determining the success of emission reduction cooperation is carbon-reduction efficiency which will directly affect the amount of resources you need to invest in a given target or the effect of carbon reduction it can produce with given resources. The designer always expects to cooperate with an efficient contract manufacturer of carbon reduction. On the one hand, it can reduce the cost of low carbon product and thus reduce the retail price, and then increase competitiveness of low carbon products. On the other hand, with the same cost of carbon reduction, it can enhance the level of carbon reduction and meet the demand of consumers with the preference for low carbon production. In practice, the contract manufacturer (i.e., Foxconn) always has more information of carbon reduction efficiency compared with the designer (i.e., Apple Inc.). Hence, we focus on the information asymmetry issue that the manufacturer has the private cost information of carbon reduction efficiency. The designer does not know the accurate value of the carbon reduction cost, but she knows the distribution of carbon reduction efficiency of the manufacturer. The information asymmetry of carbon reduction efficiency increases the decision uncertainty within the supply chain. Particularly, the disclosure of information would expose the manufacturer in an unfavorable position when contracting with the designer. Accordingly, how to induce the manufacturer to reveal his real information of carbon reduction efficiency would be an important and challenging problem.

To address the above issues, we investigate a supply chain consisting of a designer and a manufacturer. Designers (such as Apple Inc.) provide the product design scheme and choose a manufacturer (such as Foxconn) who makes products according to the requirement of the designer. To analyze the relationship between a designer and a manufacturer, we analyze the incentive strategy issue in the Stackelberg game to make sure that the designer has the initiative to induce the manufacturer with private information of carbon reduction efficiency to show its true type of carbon reduction efficiency. On the basis of parameter combination regarding differences in carbon-reduction efficiency, we propose the pooling and separating equilibrium models, and compare profits of supply chain members under different equilibrium models and the influences of the probability of high-efficiency (low-efficiency) manufacturers with regard to carbon reduction on the profits of supply chain members. We propose the following issues: (i) In the context of asymmetric information, which signaling strategy is more effective for supply chains consisting of retailers and manufacturers? (ii) In different equilibrium strategies, the probability of high-efficiency emission reduction manufacturers and the impact on efficient emission reduction manufacturers and designs. Does an increase in the probability of an efficient manufacturer means an increase in the level of emission reduction in the supply chain and an increase in the profit of manufacturers of highly efficient emission reductions? (iii) For pooling or separating equilibrium models, how does the performance of the efficient or inefficient manufacturers of emission reduction change compared to the case of information symmetry, in which model the manufacturer’s the decision-making will be distorted? (iv) For the designer, in which case is his performance optimal?

The rest of the paper is organized as follows. Section 2 surveys the relevant literature on cost information asymmetry and low-carbon supply chain, while we characterize the cost information asymmetry problem in low-carbon supply chain in Section 3. Section 4 analyzes several different cases (i.e., the pooling equilibrium and separating equilibrium cases). We present the numerical analysis in Section 5. Finally, we summarize the whole paper in Section 6.

## 2. Literature Review

We review two streams of literature pertinent to our research. The first is cost information asymmetry in supply chains and the second is about low-carbon supply chains.

A number of recent studies have investigated cost information asymmetry in supply chains. Cao et al. [14] modeled the optimal wholesale contract design problem in a dual-channel supply chain under information asymmetry and analyzed the effect of cost information asymmetry on equilibrium strategies and supply chain member profits. Regarding service cost information asymmetry, Du et al. [15] addressed contract design problems for service provider and found that the revenue-sharing contract could not solve the issue of non-contractible service quality. Huang and Yang [16] examined the optimal contract design issue when the supplier could obtain the cost information and considered the information disclosure strategies. Ma et al. [17] proposed an optimal contract for a two-level supply chain in cost information asymmetry. The authors examined the impact of CSR (corporate social responsibility) cost on CSR commitment and profits. Agrell and Bogetoft [18] addressed the contractual choice problem between centralization or delegating the investment decision in a three-level supply chain under information asymmetry. Mahadevan et al. [19] analyzed the influence of cost heterogeneity on the outsourcing strategies of a client who outsourced her service to outside service providers where the vendor kept his cost structure information, and obtained the relationship between the client mean demand and the number of service providers. Ma et al. [20] tried to design a sourcing mechanism within a supply chain with a manufacturer and suppliers subject to an emission trading scheme where the information of green degree is privately held by suppliers.

Overall, only a few of publications on cost information asymmetry have focused on the low-carbon supply chain. Compared with the pertinent literature, the present study differs as it considers the possible impact of the manufacturer’s emission reduction efficiency on the supply chain members. We point this out that when the probability gap between the high-efficiency and low-efficiency manufacturers of carbon reduction is relatively large. The two contract models (the pooling equilibrium model and the separating equilibrium model) almost have no impacts on supply chain members.

Signaling game literature is related to this study. Krishnan and Manu [21] analyzed how an incumbent retailer strategically manages its demand information under leakage within supply chain to maximize its profit. Under the framework of Krishnan and Manu [21], Kong et al. [22] demonstrated that the revenue-sharing contracts can alleviate the negative effects of information leakage within supply chain. Qin et al. [23] demonstrated the transmission mechanism of the retailer’s fairness-concern information with signaling game and analyzed the existing of the separating equilibrium and pooling equilibrium under asymmetric information. Beer et al. [24] showed that the buyer-specific investment can be used as a signal of trustworthiness and that the supply chain performance improves when the trustworthiness of suppliers can be identified. There is relatively little literature pointing to signaling game in low-carbon supply chain and it mainly focuses on non-low carbon issues research.

As we all know, massive carbon emissions is the main factor behind global warming, which exposes millions of people to life-threatening heat waves, water shortages, and coastal flooding. In 1992, the United Nations Framework Convention on Climate Change opened for signature at the Earth Summit in Rio de Janeiro, aiming to control the level of greenhouse gas (UNFCCC [25]). In 1998, the cap-and-trade policy was provided to control carbon emission at the Kyoto Protocol. Furthermore, the Copenhagen Accord in 2009, the Doha Amendment in 2012, and the Paris Agreement in 2015 together constitute the international environmental treaty aiming to control the carbon emissions and mitigate the negative influence of global warming. Under this regard, low-carbon has become a hot topic deserving everyone's attention.

The other stream of research relevant to our study is the literature on low-carbon supply chain. We refer readers to Taticchi et al. [26], Eskandarpour et al. [27], Dubey et al. [28], Reefke and Sundaram [29], and Das and Jharkharia [30] for a comprehensive review on low-carbon supply chain management. Du et al. [4] and Du et al. [5] analyzed the effects of carbon emissions and the low-carbon preference of consumers on production strategies under the cap-and-trade policy and built a production optimization model. Du et al. [15] presented the conditions in which low-carbon production was profitable. Nematollahi et al. [31] considered a supplier–retailer supply chain with the newsvendor model and compared the results of decentralized, centralized, and collaborative models. These authors found that under certain conditions, with the increment of supply chain profits, additional CSR investment and an improved CSR performance level can be achieved. Panda et al. [32] focused on a manufacturer-retailer supply chain coordination and checked profit maximizing views of channel members for CSR. They also analyzed the efficiency of a revenue sharing contract and a quantity discount contract in order to coordinate a socially responsible supply chain. Li et al. [33] addressed the outsourcing issues of production and transportation in a two-level supply chain under policies of cap-and-trade, joint cap-and-trade, and carbon tax. Park et al. [34] investigated the influences of imposing carbon costs on a supply chain and social welfare in three competitive settings (monopoly, monopoly competition with symmetric market share, and monopolistic competition with asymmetric market share). Wang et al. [35] considered the joint carbon reduction problem in a supply chain within the retailer dominant and power balanced cases. Bazan et al. [36] proposed two models, namely, classical and VMI-CS (vendor managed inventory with consignment stock) coordination models, for a closed-loop supply chain to remanufacture used items and they compared the results with through numerical analysis. Different from the above literature, we focus on a low-carbon supply chain consisting of a designer and a manufacturer and examine the designer’s partner choice issue in the low-carbon context.

The contributions of this study are as follows. The aforementioned studies focused on production cost information asymmetry. In this study, we consider that the low-carbon preference of consumers (Echeverrı´a et al. [37], Loo et al. [38], and Vecchio and Annunziata [39]) significantly drives supply chain to reduce carbon emissions as per the Paris Agreement. Thus, the efficiency of carbon reduction becomes a major factor influencing the decision-making of supply chain members. The analysis of the information asymmetry of emissions reduction efficiency helps lessen its negative influence on supply chain performance. In addition, we examine the effects of the probability of the high(low)-efficiency manufacturers in terms of emission reduction.

## 3. Preliminaries and the Basic Model

### 3.1. Preliminaries

We assume a low-carbon supply chain is consisting of a designer (denoted as *d*) and a manufacturer (denoted as *m*). For ease of elaboration, we use ‘He’ to denote the manufacturer and ‘She’ to denote the designer. The designer provides its design scheme to the manufacturer. To cater consumers’ preference for low-carbon products, the manufacturer invests on carbon reduction and sells its low-carbon products to the end market. Suppose that the efficiency type of carbon emission demonstrated by the investment coefficient of carbon reduction is his private information of the manufacturer. The designer does not know the actual efficiency type carbon reduction of the manufacturer, but knows the probability distribution that the manufacturer belongs to the high-efficiency type (denoted by the subscript ‘H’) is θ, and that the manufacturer belongs to the low-efficiency type (denoted by the subscript ‘L’) is 1−θ, where the probability θ is in fact uniformly distributed on the interval [0,1]. The above information is considered common knowledge of both the designer and manufacturer. The information asymmetry regarding the information efficiency seriously affects the optimal decision of supply chain members. Therefore, it is vital to design reasonable mechanism to address the information asymmetry issue to induce the manufacturer to report his actual efficiency type of carbon reduction efficiency.

We assume that the low carbon product obtains more market demand than the non-low carbon products due to the low carbon preference of consumers. More carbon reduction leads to more market demand. In line with related literature (Du et al. [5], Wang et al. [35]), we present the demand function in a linear form as follows:

(1) D=α−βp+γe

where α is the base demand market size, β is the price-sensitive coefficient, p is the retail price, γ measures the influence of the carbon reduction on demand, and e is the carbon reduction level. The demand function decreases in the retail price and increase in the carbon reduction level.

We assume that the investment cost of carbon reduction is a one-time investment C(e), and that it is an increasing convex function of e. Considering the effect of diminishing marginal return on the environment expenditure, we thus use a quadratic function of the carbon reduction level to represent the carbon reduction investment, namely, $C(e)=ke^{2}/2$, where k is the investment coefficient of carbon reduction. For using such a quadratic function to indicate this kind of expenditure we may also refer to Nordhaus [40], Liuabc [41], and D’Aspremont and Jacquemin [42]. Let H denote the high-efficiency manufacturer of carbon reduction and L denote the low-efficiency manufacturer.

The designer chooses the two-part tariff contract and presents the parameters combination (w,T), w denotes the wholesale price, and T is the lump-sum transfer payment to maximize its profit. After getting the designer’s decision information, the manufacturer maximizes his profit by determining the decision variables p and e.

The profit functions of both the designer and the manufacturer can be given respectively as

(2) $$\pi_{d}=\pi_{d}(w,T)=(w-c)(\alpha -\beta p+\gamma e)+T,$$

where the first part on the right-hand side is the gross profit from providing the design scheme and T is a lump-sum transfer payment;

(3) $$\pi_{m}=\pi_{m}(p,e)=(p-w)(\alpha -\beta p+\gamma e)-ke^{2}/2-T$$

where the first part represents the gross profit obtained by selling the low-carbon product to the end-market, the second part is the investment on carbon reduction, and T is the fixed expenditure paid to the designer.

The notations used in this paper are summarized in the Table 1 below.

In the following section, we analyze the information symmetry case as the first-best solutions for low-carbon supply chain.

### 3.2. Basic Model with Information Symmetry

In the case of information symmetry, the designer and manufacturer are fully informed of the carbon reduction. They are also aware that their partner knows the information. As the manufacturer cannot hide his real information of carbon reduction efficiency under information symmetry, it leads to a situation where the incentive compatibility constraints are no longer satisfied while leaving the participation constraints in place. The planning can be stated as

(4) $$\pi_{d}=\pi_{d}(w,T)=(w-c)(\alpha -\beta p+\gamma e)+T,$$

subject to

(5) $$e,p\in \arg\;\max(p-w)(\alpha -\beta p+\gamma e)\;-ke^{2}/2-T$$

(6) $$\max\pi_{m}=(p-w)(\alpha -\beta p+\gamma e)-ke^{2}/2-T\geq U_{0}$$

The Formula (4) is the object function of the designer. Equation (5) is the incentive compatibility constraint, which makes sure that the manufacturer chooses the behavior that maximizes his profit. $U_{0}$ is the manufacturer’s reserved profit. It is the lowest profit he can accept. Even if he does not sign the contract offered by the designer, he could also obtain the profit as $U_{0}$. Whose specific value can be derived from historical profit or the average profit of similar companies. Equation (6) is the participation constraint, which guarantees that the manufacturer could obtain his reserve profit when he accepts the contract. Therefore, the participation constraint must be satisfied.

By solving the mathematical planning composed by Equations (4) and (6), we obtain the optimal wholesale price, carbon emission reduction level, retail price, the profits of designer and manufacturer, respectively.

(7) $$w=c,\;e=\frac{\gamma (\alpha -c\beta )}{2k\beta -\gamma^{2}},\;p=\frac{k(\alpha +c\beta )-c\gamma^{2}}{2k\beta -\gamma^{2}},\;\pi_{d}=T=\frac{k{\left(\alpha -\beta c\right)}^{2}}{4k\beta -2\gamma^{2}}-U_{0},\;\pi_{m}=U_{0}$$

Similar to the classical model in principal-agent issues (Laffont and Martimort [43]), the designer as the principal obtains all profit created by the supply chain system except the reserved profit of the manufacturer at the wholesale price c.

**Proposition** **1.** *The lump-sum fee in the information symmetry case decreases in the coefficient of carbon reduction investment*k. *(See the proof in the Appendix A.1)*.

It is easy to get the result. When the coefficient of carbon reduction investment is high, it indicates that the manufacturer has a poor profitability. Thus, the designer charges a lower lump-sum fee for the low-efficient manufacturer of carbon reduction. It leads to the possibility that the high-efficiency type manufacturer with a lower k would mimic the low-efficiency type to lower his lump-sum fee, especially in the case where there is information asymmetry with regard to k. To address the problem, the designer should develop a proper mechanism to avoid this situation.

## 4. Information Asymmetry

**Figure 1:**
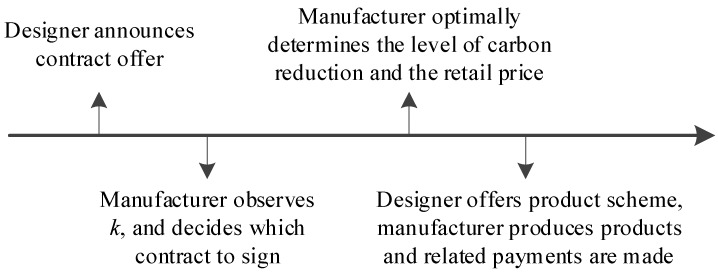
Decision sequence time under information asymmetry.

Under information asymmetry, the manufacturer keeps the private information about his carbon reduction efficiency type. However, the designer only knows the manufacturer’s probability distribution of the carbon reduction efficiency type. As the carbon-reduction efficiency information asymmetry, this problem is an adverse selection problem. The agent-principal theory is a traditional method to solve this kind of problem (Cao et al. [14], Agrell and Bogetoft [18], Mahadevan et al. [19], and Laffont and Martimort [43]). To maximize her profit, the designer has two choices. One offers the same contract to manufacturers with different efficiency types of carbon reduction (the pooling equilibrium model). The other one offers two different contracts aimed at two types of manufacturers; it is up to the manufacturers which contract to select (the separating equilibrium model). The decision sequence is shown in Figure 1. In this section, we first analyze the pooling equilibrium model and then investigate the separating equilibrium model.

### 4.1. Pooling Equilibrium Model under Information Asymmetry of Carbon Reduction Efficiency

In the pooling equilibrium model, neither the designer nor the leader distinguishes the manufacturer’s carbon reduction types and offers the same contract, i.e., (w,T). Given the contract information, the manufacturer as the follower chooses to accept or decline the offer of the designer after obtaining his information of carbon reduction efficiency. When the manufacturer chooses to accept the offer, then he will determine the retail price and level of carbon reduction on the basis of his private information. In the pooling equilibrium model, the planning can be given by

(8) $$\pi_{d}=\pi_{d}(w,T)=\theta [(w-c)(\alpha -\beta p_{H}+\gamma e_{H})+T]+(1-\theta )[(w-c)(\alpha -\beta p_{L}+\gamma e_{L})+T]$$

Subject to

(9) $$p_{L},e_{L}\in \arg\max(p_{L}-w)(\alpha -\beta p_{L}+\gamma e_{L})-k_{L}e_{L}^{2}/2-T$$

(10) $$p_{H},e_{H}\in \arg\max(p_{H}-w)(\alpha -\beta p_{H}+\gamma e_{H})-ke_{H}^{2}/2-T$$

(11) $$\pi_{L}=(p_{L}-w)(\alpha -\beta p_{L}+\gamma e_{L})-k_{L}e_{L}^{2}/2-T\geq U_{0}$$

(12) $$\pi_{H}=(p_{H}-w)(\alpha -\beta p_{H}+\gamma e_{H})-k_{H}e_{H}^{2}/2-T\geq U_{0}$$

Equation (8) is the expected profit of the designer when the efficiency type of the manufacturer is either high or low with the probability of θ and 1−θ, respectively. Equations (9) and (10) are incentive compatibility constraints, whereas the Equations (11) and (12) are participation constraints.

From incentive compatibility constraints (9) and (10), we have

(13) $$p_{i}=\frac{k_{i}\alpha +w(k_{i}\beta -\gamma^{2})}{2k_{i}\beta -\gamma^{2}},\;e_{i}=\frac{\gamma (\alpha -\beta w)}{2k_{i}\beta -\gamma^{2}},\;i\in \left(L,H\right)$$

Substituting Equations (13) into (11) and (12), we can obtain

(14) $$\pi_{i}=\frac{k_{i}{\left(\alpha -w\beta \right)}^{2}}{4k_{i}\beta -2\gamma^{2}}-T,\;i\in \left(L,H\right)$$

It is easy to see that the profit of the manufacturer decreases in the investment coefficient of carbon reduction. We then have $\pi_{H}>\pi_{L}$. Accordingly, when the low-efficiency manufacturer of carbon reduction satisfies the participation constraint, the high-efficiency manufacturer can meet the same constraint. Therefore, for the constraints (11) and (12), we only need to retain the constraint (11). It also needs to note that the designer may also adjust the parameter combination to equalize the expected profit of the low-efficiency manufacturer with his reserved profit, i.e., the constraint (11) is binding. Thus, we have

(15) $$T=\frac{k_{L}{\left(\alpha -\beta w\right)}^{2}}{4k_{L}\beta -2\gamma^{2}}-U_{0}$$

**Proposition** **2.** *In the pooling equilibrium model under information asymmetry, the low-efficiency manufacturer with low-carbon reduction retains his reserved profit, and the high-efficiency manufacturer obtains information rent that increases in*$k_{L}-k_{H}$. *(See the proof in the Appendix A.2)*.

Note that here we define the difference $\Delta =\pi_{H}-\pi_{m}$ as the information rent of an efficient manufacturer with carbon reduction. The proposition characterizes that the low-efficiency type manufacturer of carbon reduction only gets his reserved profit. The profit of the high-efficiency type manufacturer increases in the efficiency difference of carbon reduction between the high-efficiency type and low-efficiency type. Under information asymmetry of carbon reduction efficiency, the low-efficiency manufacturer obtains only his reserved profit. By contrast, the high-efficiency manufacturer does not only obtain information rent, but also his reserved profit due to his private carbon reduction efficiency information. The designer also needs to pay the high-efficiency manufacturer additional information rent when the efficiency gap of carbon reduction between the high-efficiency and low-efficiency increases. In other words, reducing the gap of carbon reduction efficiencies helps to compress the information rent paid to the high-efficiency manufacturer. Therefore, to reduce the information rent, it would be sensible for the designer to classify the manufacturers or refer to the third party’s classification results when choosing the potential partners. Confining the manufacturers in one class would diminish the efficiency gap in carbon reduction among potential partners, thereby reducing supply chain risk and improving performance.

We substitute the retail price and carbon-reduction level into the Formula (8). The expected profit function of the designer can then be rewritten as

(16) $$\max\pi_{d}=\theta [\frac{k_{H}(w-c)\beta (\alpha -w\beta )}{2k_{H}\beta -\gamma^{2}}+\frac{k_{L}{\left(\alpha -w\beta \right)}^{2}}{4k_{L}\beta -2\gamma^{2}}-U_{0}]+(1-\theta )[\frac{k_{L}(w-c)\beta (\alpha -w\beta )}{2k_{L}\beta -\gamma^{2}}+\frac{k_{L}{\left(\alpha -w\beta \right)}^{2}}{4k_{L}\beta -2\gamma^{2}}-U_{0}]$$

The first order condition of (16) with respect to the wholesale price w can be given by

(17) $$\frac{\partial \pi_{d}}{\partial w}=\frac{\beta \{-(\alpha +(c-2w)\beta \gamma^{2}\theta k_{H}+k_{L}[\gamma^{2}(\alpha \theta +\beta (w+c(-1+\theta )-2w\theta ))+2(c-w)\beta^{2}k_{H}]\}}{\left(\gamma^{2}-2\beta k_{L})(\gamma^{2}-2\beta k_{H}\right)}=0$$

Thus, we have

(18) $$w=\frac{k_{L}[2c\beta^{2}k_{H}-\gamma^{2}(c\beta (1-\theta )-\alpha \theta )]-(\alpha +c\beta )\gamma^{2}\theta k_{H}}{\beta k_{L}[2\beta k_{H}-\gamma^{2}(1-2\theta )]-2\beta \gamma^{2}\theta k_{H}}$$

Substituting Equation (18) into Equations (13) and (15) gives

(19) $$T=\frac{{\left(\alpha -c\beta \right)}^{2}k_{L}{\left[\gamma^{2}\theta k_{H}+k_{L}(\gamma^{2}(1-\theta )-2\beta k_{H})\right]}^{2}}{2(2\beta k_{L}-\gamma^{2}){\left[2\gamma^{2}\theta k_{H}-k_{L}(\gamma^{2}(2\theta -1)+2\beta k_{H})\right]}^{2}}-U_{0}$$

(20) $$e_{H}=\frac{\gamma (\alpha -c\beta )[k_{L}(2\beta k_{H}-\gamma^{2}(1-\theta ))-\gamma^{2}\theta k_{H}]}{\left(2\beta k_{H}-\gamma^{2})[k_{L}(2\beta k_{H}-\gamma^{2}(1-2\theta ))-2\gamma^{2}\theta k_{H}\right]}$$

(21) $$e_{L}=\frac{\gamma (\alpha -c\beta )[k_{L}(2\beta k_{H}-\gamma^{2}(1-\theta ))-\gamma^{2}\theta k_{H}]}{\left(2\beta k_{L}-\gamma^{2})[k_{L}(2\beta k_{H}-\gamma^{2}(1-2\theta ))-2\gamma^{2}\theta k_{H}\right]}$$

**Proposition** **3.** *Comparing the optimal decisions in the pooling equilibrium and full information, we have results as follows (I)*$w^{*}<w_{p}$, (II) $p_{H}^{*}<p_{PH}$, (III) $e_{L}^{*}>e_{PL}$, (IV) $p_{L}^{*}<p_{PL}$, (V) $e_{H}^{*}>e_{PH}$.

The proof is straightforward. The proposition characterizes that the carbon reduction level is downward distorted, and the retail price as well as the wholesale price are upward distorted for the high and low-efficiency manufacturers in the pooling equilibrium model. Due to the information asymmetry regarding the carbon reduction efficiency information, it induces the manufacturer to overprice and under determine the level of carbon reduction. Both the over-price and the lower level of carbon reduction result in an insufficient demand, which will lead to the decline in profit of the supply chain.

### 4.2. Separating Equilibrium Model under the Information Asymmetry of Carbon Reduction Efficiency

In the pooling equilibrium model, the designer does not distinguish the carbon reduction efficiency types, which may not be of any help to address the information asymmetry issues. Therefore, to reduce the losses caused by information asymmetry, the designer focuses on the differences of the carbon reduction efficiency. In the separating equilibrium model, to distinguish manufacturers with different efficiency of carbon reduction, the designer offers manufacturers with two different contract options, i.e., $\left(w_{L},T_{L}\right)$ and $\left(w_{H},T_{H}\right)$.

In this stage of this model, the designer presents two contract options with two parameter combinations for the manufacturer to either select or do nothing; In the second stage, after trading off the effects of the two contract options on his profit on the basis of his private carbon reduction efficiency information, the manufacturer selects a suitable contract, or chooses not to trade with the designer. When the manufacturer either chooses a contract, which also reflects his carbon reduction efficiency information, he needs to determine the retail price and carbon reduction level to maximize his profit.

Accordingly, under the information asymmetry of carbon reduction, the planning of separating equilibrium model planning can be written as

(22) $$\max\pi_{d}=\theta [(w_{H}-c)(\alpha -\beta p_{H}+\gamma e_{H})+T_{H}]+(1-\theta )[(w_{L}-c)(\alpha -\beta p_{L}+\gamma e_{L})+T_{L}]$$

subject to

(23) $$\begin{array}{l} \pi_{LL}=(p_{L}-w_{L})(\alpha -\beta p_{L}+\gamma e_{L})-k_{L}e_{L}^{2}/2-T_{L}\geq \\ \pi_{LH}=(p_{LH}-w_{H})(\alpha -\beta p_{LH}+\gamma e_{LH})-k_{L}e_{LH}^{2}/2-T_{H} \end{array}$$

(24) $$\begin{array}{l} \pi_{HH}=(p_{H}-w_{H})(\alpha -\beta p_{H}+\gamma e_{H})-k_{H}e_{H}^{2}/2-T_{H}\geq \\ \pi_{HL}=(p_{HL}-w_{L})(\alpha -\beta p_{HL}+\gamma e_{HL})-k_{H}e_{HL}^{2}/2-T_{L} \end{array}$$

(25) $$\pi_{LL}=(p_{L}-w)(\alpha -\beta p_{L}+\gamma e_{L})-k_{L}e_{L}^{2}/2-T_{L}\geq U_{0}$$

(26) $$\pi_{HH}=(p_{H}-w)(\alpha -\beta p_{H}+\gamma e_{H})-k_{H}e_{H}^{2}/2-T_{H}\geq U_{0}$$

The incentive compatibility constraints (IC) (23) and (24) apply the revelation principle (Chen [44] and Laffont and Martimort [43]) that allows the high-efficiency manufacturer to obtain a higher profit $\pi_{HH}$ when choosing $\left(w_{H},T_{H}\right)$ than the profit $\pi_{HL}$ when choosing $\left(w_{L},T_{L}\right)$. Similarly, the constraint (23) shows that selecting the contract $\left(w_{L},T_{L}\right)$ benefits the low-efficiency manufacturer. Equations (25) and (26) are participation constraints, which guarantees that the manufacturer may join the low-carbon supply chain and acquire his reserved profit.

In this Stackelberg game model, the manufacturer sets the retail price and the carbon reduction level based on its own private carbon reduction efficiency information with backward induction, given the parameter combination of the designer.

Let $\partial \pi_{i}/\partial e_{i}=0$, $\partial \pi_{i}/\partial p_{i}=0$, $\partial \pi_{ij}/\partial p_{ij}=0$, $\partial \pi_{ij}/\partial e_{ij}=0$, thus we have

(27) $$p_{i}=\frac{k_{i}\alpha +w_{i}(k_{i}\beta -\gamma^{2})}{2k_{i}\beta -\gamma^{2}},\;e_{i}=\frac{\gamma (\alpha -\beta w_{i})}{2k_{i}\beta -\gamma^{2}},\;i\in (L,H)$$

(28) $$p_{ij}=\frac{k_{i}\alpha +w_{j}(k_{i}\beta -\gamma^{2})}{2k_{i}\beta -\gamma^{2}},\;e_{ij}=\frac{\gamma (\alpha -\beta w_{j})}{2k_{i}\beta -\gamma^{2}},\;i,j\in (L,H)$$

To maximize his profit, the high-efficiency manufacturer of carbon reduction imitates the low-efficiency to obtain a lower wholesale price from the designer. However, the low-efficiency one does not need to imitate the high-efficiency. Therefore, it only needs to keep the constraint Equation (24) between the incentive compatibility constraint Equations (23) and (24) in the planning, which automatically satisfies the constraint (23).

**Proposition** **4.** *The incentive compatibility constraint of the low-efficiency manufacturer Equation (23) is a redundant constraint, as presented in the Appendix A.3*.

In the separating equilibrium model, the constraints Equations (23) and (26) are binding, as the designer can adjust the parameter combination to control the manufacturer’s profit.

From the Equation (26), we get

(29) $$T_{L}=(p_{L}-w)(\alpha -\beta p_{L}+\gamma e_{L})-k_{L}e_{L}^{2}/2-U_{0}=\frac{k_{L}{\left(\alpha -w_{L}\beta \right)}^{2}}{4k_{L}\beta -2\gamma^{2}}-U_{0}$$

(30) $$T_{H}=\frac{k_{H}{\left(\alpha -\beta w_{L}\right)}^{2}}{4k_{H}\beta -2\gamma^{2}}-\frac{k_{H}{\left(\alpha -\beta w_{H}\right)}^{2}}{4k_{H}\beta -2\gamma^{2}}+T_{L}$$

Substituting Equations (29) and (30) into Equation (22), we obtain the first level conditions with respect to $w_{H}$ and $w_{L}$ are

(31) $$\frac{\partial \pi_{d}}{\partial w_{L}}=\frac{\beta \{\gamma^{2}\theta k_{H}(-\alpha +\beta w_{L})+k_{L}[2\beta^{2}(1-\theta )k_{H}(c-w_{L})+\gamma^{2}\left(c\beta (-1+\theta )+\alpha \theta +(\beta -2\beta \theta )w_{L}\right)]\}}{\left(\gamma^{2}-2\beta k_{L})(\gamma^{2}-2\beta k_{H}\right)}=0$$

(32) $$\frac{\partial \pi_{d}}{\partial w_{H}}=\frac{-2\beta^{3}k_{L}k_{H}(-2c\theta +2\theta w_{H})+\beta \gamma^{2}\theta k_{H}[\beta w_{H}+\beta (-2c+w_{H})]}{2(\gamma^{2}-2\beta k_{L})(\gamma^{2}-2\beta k_{H})}=0$$

We solve the equation set consisting of Equations (31) and (32), and obtain

(33) $$w_{H}=c$$

(34) $$w_{L}=\frac{\alpha \theta k_{H}\gamma^{2}+k_{L}\{\gamma^{2}[c\beta (1-\theta )-\alpha \theta ]-2c\beta^{2}(1-\theta )k_{H}\}}{\beta \theta k_{H}\gamma^{2}+\beta k_{L}[\gamma^{2}(1-2\theta )-2\beta (1-\theta )k_{H}]}$$

Substituting Equation (33) into Equation (27), we have

(35) $$p_{H}=\frac{k_{H}(\alpha +c\beta )-c\gamma^{2}}{2\beta k_{H}-\gamma^{2}},\;e_{H}=\frac{(\alpha -c\beta )\gamma }{2\beta k_{H}-\gamma^{2}}$$

**Proposition** **5.** *The decision of the high-efficiency manufacturer in the separating equilibrium model is his first-best result.*

By comparing Equations (7) and (35), we can see that the decision of the high-efficiency manufacturer is not distorted by the information asymmetry of carbon reduction efficiency. This finding is in line with classic theory.

**Proposition** **6.** *The designer sells her design scheme to the high-efficiency manufacturer of carbon reduction at the wholesale price*c. *The wholesaler price of the low-efficiency manufacturer increases in his probability*1−θ*and decreases in his investment coefficient of carbon reduction*$k_{L}$.

By taking derivatives, it is easy to get the above conclusion. The designer provides raw materials to the high-efficiency manufacturer at her production cost and only obtains the fixed income from him. Proposition 5 indicates that the wholesale price increases in the high-efficiency manufacturer’s probability, and decreases in the probability of the low-efficiency manufacturer. This phenomenon can be explained by further cooperation with the high-efficiency manufacturer when they are prevalent in the market. Additionally, in the same scenario, the designer should reduce the cooperation with the low-efficiency manufacturer by setting a higher wholesale price.

The increasing in the carbon reduction investment coefficient of the low-efficiency manufacturer indicates a decrease in the gap between the high and low-efficiency manufacturer. Correspondingly, the boundary between manufacturers of different efficiency types is obscured. In such a case, discriminatory practices for the two types of manufacturers become unnecessary.

Substituting the Equations (33) and (34) into Equations (29) and (30) respectively, we thus obtain

(36) $$T_{L}=\frac{{\left(\alpha -c\beta \right)}^{2}{\left(1-\theta \right)}^{2}{\left(2\beta k_{H}-\gamma^{2}\right)}^{2}k_{L}^{3}}{2(2\beta k_{L}-\gamma^{2}){\left\{\gamma^{2}\theta k_{H}+k_{L}[\gamma^{2}(1-2\theta )-2\beta (1-\theta )k_{H}]\right\}}^{2}}-U_{0}$$

(37) $$T_{H}=\frac{k_{H}{\left(\alpha -c\beta \right)}^{2}}{4\beta k_{H}-2\gamma^{2}}+\frac{k_{H}k_{L}^{2}{\left(\alpha -c\beta \right)}^{2}{\left(1-\theta \right)}^{2}\left(\gamma^{2}-2\beta k_{H}\right)}{2{\left\{\gamma^{2}\theta k_{H}+k_{L}\left[\gamma^{2}\left(1-2\theta \right)-2\beta \left(1-\theta \right)k_{H}\right]\right\}}^{2}}\;\;+\frac{{\left(\alpha -c\beta \right)}^{2}{\left(1-\theta \right)}^{2}{\left(\gamma^{2}-2\beta k_{H}\right)}^{2}k_{L}^{3}}{2\left(2\beta k_{L}-r^{2}\right){\left\{\gamma^{2}\theta k_{H}+k_{L}\left[\gamma^{2}\left(1-2\theta \right)-2\beta \left(1-\theta \right)k_{H}\right]\right\}}^{2}}$$

## 5. Numerical Analysis

In view of the complexity of the above analytic expressions, this section explores the effects of different parameters on the relevant decision variables and profits with the numerical analysis. Specific values for the relevant parameters are as follows: α=100, β=1, $k_{H}=1000$, $k_{L}=2000$, γ=20, c=10, $U_{0}=1000$.

### 5.1. Influence of θ on the Profits of the Designer and Manufacturer

**Figure 2:**
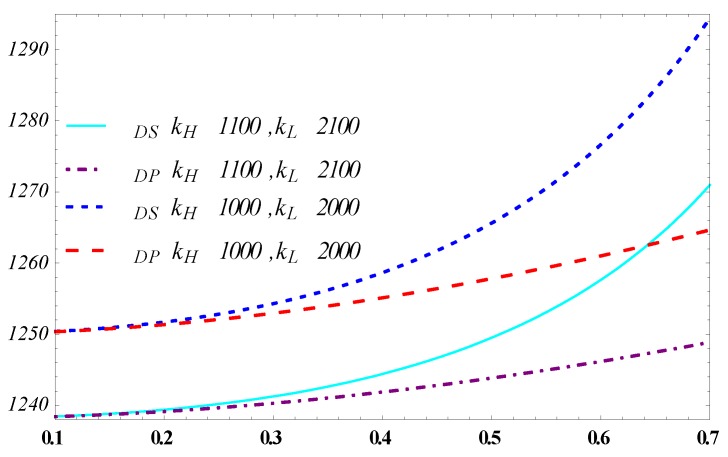
Influence of θ on the designer’s profit.

**Figure 3:**
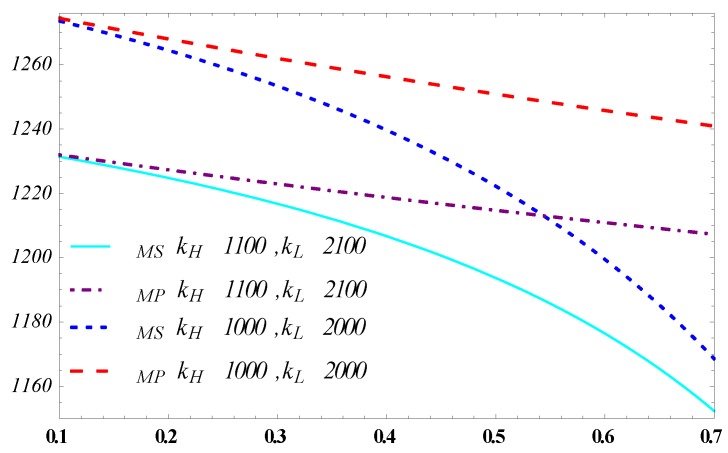
Influence of θ on the high-efficiency manufacturer of carbon reduction.

In Figure 2 and Figure 3, under pooling and separating equilibrium models, the profit of the designer increases with the probability of the high-efficiency manufacturer. However, the profit of the high-efficiency manufacturer decreases with this probability. This trend shows how likely a manufacturer, with a high-efficiency type of carbon reduction, directly affects the information rent. A low probability of a manufacturer to be of the high-efficiency type more likely leads to a high information rent, which is due to the carbon reduction efficiency advantage over a low-efficiency. Thus, the designer needs to pay more for a high-efficiency manufacturer. When the probability is relatively high, which may mean that the carbon reduction in the whole industry is improved, the information rent from private information will be largely compressed. This shows that the designer should participate in the process of carbon reduction technology updating and diffuse related new information of carbon reduction technology among supply chain members by holding technology forums of manufacturers with systematic thinking rather than do nothing.

From the perspective that θ increases from zero to one on the horizontal axis it can be explained that the advanced technology is developed initially by very few manufacturers, and hiding the real information of carbon reduction efficiency is relatively easy, which corresponds to the lower area on the horizontal axis of θ. With the diffusion of the advanced technology in the industry, more and more manufacturers grasp this capability and keep the cost of carbon reduction at a low level, which responds to the higher area on the axis of *θ*. It also results in the difficulty of hiding his private information of carbon reduction efficiency for the high-efficiency manufacturer. In the separating equilibrium model, when *θ* is close to one—i.e., all the potential partners of the designer obtain this technology—the information rent will approach to zero. While in the pooling equilibrium model, even if most of the potential partners of the designer improve their carbon reduction efficiency, the high-efficiency manufacturer will still obtain information rent due to the parameter combination of the designer. In this case, the rational designer should offer different parameter combinations for manufactures to select.

Comparing the separating and the pooling equilibrium model shows that the first model can increase the profit for the designer, whereas the pooling equilibrium model increases profits for the manufacturer. This results from the parameter combination concerning to the efficiency type of carbon reduction between the separating and pooling equilibrium models. The separating equilibrium model presents different contract parameters concerning the efficiency of carbon reduction, which squeezes down the information rent of the high-efficiency. When *θ* nears one, the profit of the designer increases sharply in the separating equilibrium model, but increases smoothly in the pooling equilibrium model.

What needs to be pointed out is that when *θ* is in the lower range (θ<0.25), i.e., the high-efficiency manufacturer has a lower probability, the profits of the manufacturer or designer in the pooling equilibrium model and separating equilibrium model have few differences. The pooling equilibrium contract is simpler in the form than the separating equilibrium contract. Besides, it could reduce the cost for determining reasonable parameters in the separating equilibrium. Therefore, for the designer, selecting the pooling equilibrium is more suitable when the value of *θ* is small. Therefore, selecting the pooling equilibrium is more suitable for the designer when the value of θ is small.

### 5.2. The Impact of k on the Level of Carbon Reduction

**Figure 4:**
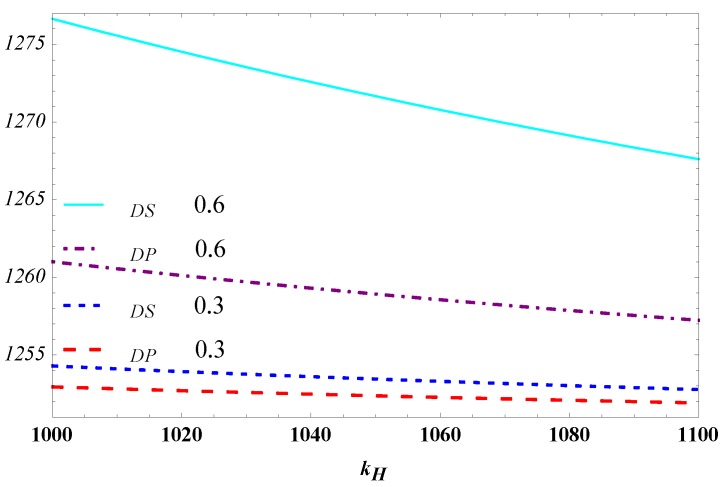
Influence of $k_{H}$ on the designer’s profit.

**Figure 5:**
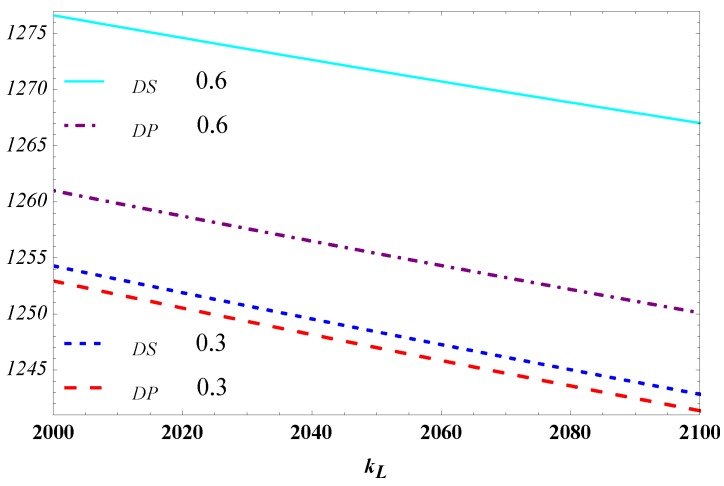
Influence of $k_{L}$ on the designer’s profit.

Figure 4 and Figure 5 demonstrate that the high efficiency and low efficiency impact on the designer profit respectively when θ in a high state and in a low state (θ=0.3 and θ=0.6). The designer’s profit decreases in the efficiency of carbon reduction which is in line with the previous analysis. What is interesting is that the gap between the separating equilibrium and the pooling equilibrium expands with the increase of the L type manufacturer’s coefficient of carbon reduction in Figure 4 while shrinks with $k_{H}$. Whereas the parameter θ positively affects the designer’s profit, the profit gap between the separating equilibrium and pooling equilibrium is larger when θ=0.6 than when θ=0.3.

This indicates that especially in the case where the high-efficiency type manufacturer has a high probability, the advantage of the separating equilibrium strategy against the pooling equilibrium strategy will be obvious when the efficiency difference of carbon reduction between the H and L type manufacturers is big; instead, it will cut back.

### 5.3. Influence of θ on the Carbon Reduction Level

**Figure 6:**
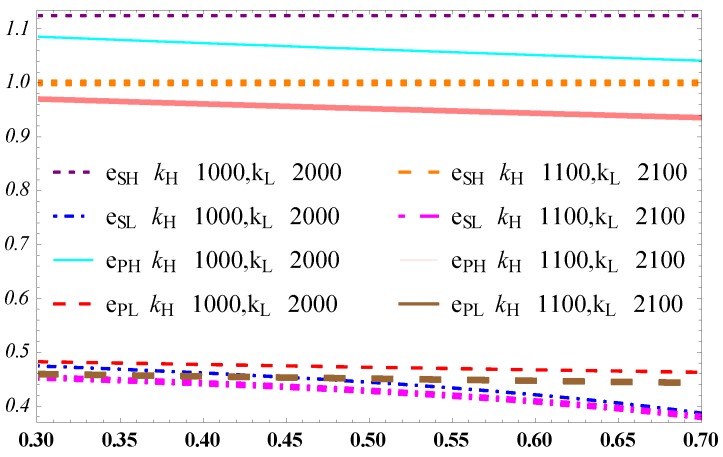
Influence of θ on the level of carbon reduction.

In Figure 6, the carbon reduction level of the high-efficiency manufacturer is higher than that of the designer both in the separating equilibrium and pooling equilibrium. It is in line with our expectation. This highlights the important of the high-efficient manufacturer of carbon reduction. The more of this kind of high-efficiency manufacturers there are in a society, the greater the effect they can have in countering climate change.

Besides, the carbon reduction level decreases in θ, which corresponds to the effects of θ on the high-efficiency type manufacturer’ profit in Figure 3. This contradicts our intuition that when the high-efficient manufacturer has a higher probability in the market, the average cost of carbon reduction would be reduced accordingly. This would correspondingly increase the level of carbon reduction. The anti-intuition phenomenon results from the fact that the designer’s profit increase in θ. In other words, the designer as the principal grabs too much profit of the supply chain with increasing θ. This leads to a shortage of resources invested in carbon reduction and the decrease of the level of carbon reduction both in the separating equilibrium and pooling equilibrium. For increasing the level of carbon reduction, the designer should control her ‘greediness’ for profit and leave more profit for the manufacturer to improve carbon reduction.

Figure 6 also indicates that the high-efficiency type of manufacturer has a higher carbon reduction level in the separating equilibrium model than in the pooling equilibrium model. This result reveals the positive role of the separating equilibrium model in reducing emission for high-efficiency manufacturers. By contrast, the low-efficiency manufacturer has a high carbon reduction level in the pooling equilibrium, but a low level of carbon reduction in the separating equilibrium. This result may be explained by the low wholesale price of the designer in the pooling equilibrium, which may encourage the low-efficiency manufacturer to reduce carbon emissions. In the separating equilibrium, the designer should offer ‘discriminatory’ wholesale prices depending on the efficiency type of carbon reduction and the low-efficiency receives a high wholesale price. When *θ* is close to one, the level of carbon reduction of the low-efficiency manufacturer will approach to zero in the separating equilibrium. This phenomenon shows that in a market where high-efficiency manufacturers prevail, the high-efficiency manufacturer has an ‘adverse effect’ on the low-efficiency manufacturer in carbon reduction.

## 6. Conclusions

This paper investigates the impacts of parameter combinations of the product designer on the operation and decision in supply chain, in which the carbon emission efficiency is a private information of manufacturer, that is, information asymmetry occurs in the supply chain. In order to induce the manufacturer to demonstrate his true information regarding the efficiency of carbon reduction, the two-part tariff contracts are designed with two models. One is the pooling equilibrium model, in which the designer set one contract (w,T) regardless of the manufacturer’s carbon reduction type. While the other one is the separating equilibrium model, in which two contracts ($\left(w_{H},T_{H}\right)$, $\left(w_{L},T_{L}\right)$) are provided by the designer in terms of the different types of manufacturer about carbon emission reduction (high-efficiency, low-efficiency). Finally, some numerical examples are provided to illustrate the equilibrium results, and the conclusions are summarized as follows: (i) When the probability of the high-efficiency manufacturer chosen is large enough, the separating equilibrium model could bring more profit for the principal and the profit increases with the probability. The result reveals that when the firm selects a partner facing with a high probability of high-efficiency partner, the separating model is his best choice, that is, the firm should design two different contracts according to the different types of partners. (ii) When the probability of high-efficiency manufacturer is within the low range, the separating equilibrium model and the pooling equilibrium model will not obviously affect the principal’s profit. While considering the simplicity of the pooling equilibrium model, the principal would choose the pooling equilibrium model. The result demonstrates that when there is a low probability of a high-efficiency partner, the firm can freely choose a separating model or pooling model. In fact, the pooling model is likely to be favored because of its simplicity. (iii) The high-efficiency manufacturer will hold a higher level of carbon reduction while the low-efficiency manufacturer has a lower level of carbon reduction in the pooling equilibrium model. The separating equilibrium model results in the first-best decision of the high-efficiency manufacturer, whereas the pooling equilibrium model can distort the high-efficiency manufacturer’s decision.

Nevertheless, this work is limited by its sole analysis of information asymmetry issues under the two-part tariff contract. Thus, whether our findings also suit other contracts needs to be investigated. In addition, this study focuses only on carbon reduction efficiency information, when other important information may also be kept private by the manufacturer. Such information includes base demand market size and benefits caused by carbon reduction which also deserve their attention. Furthermore, as countries become more and more concerned about carbon emissions, many low-carbon policies have been imposed, such as, carbon trading, carbon tax, and carbon subsidy. The low-carbon policy impacts on supply chain performance with carbon information asymmetrically, which is also an interesting research orientation.

## Figures and Tables

**Table 1:** Notations summary.

**Notations**	**Description**
α	The base demand market size
β	The price-sensitive coefficient
γ	The influence of carbon reduction level on the demand
k	Investment coefficient of carbon reduction
H	High-efficiency manufacturer of carbon reduction
L	Low-efficiency manufacturer of carbon reduction
θ	The probability that the manufacturer belongs to the high-efficiency
**Decision Variables**	**Description**
p	Retail price per unit product of manufacturer
w	Wholesale price per unit product of designer
e	Carbon emission level of manufacturer
T	The fixed payment charged by designer

## Appendix A

### Appendix A.1. Proof for Proposition 1

As $\pi_{ij}$ decreases in $k_{i}$, we thus have as follows

$$\pi_{HL}=\frac{k_{H}{\left(\alpha -w_{L}\beta \right)}^{2}}{4k_{H}\beta -2\gamma^{2}}-T_{L}>\pi_{LL}=\frac{k_{L}{\left(\alpha -w_{L}\beta \right)}^{2}}{4k_{L}\beta -2\gamma^{2}}-T_{L}$$

From Equations (24) and (26), $\pi_{HH}\geq \pi_{HL}$, $\pi_{LL}\geq U_{0}$. As a result, we have $\pi_{HH}>U_{0}$. Therefore, when we have $\pi_{LL}\geq U_{0}$, the constraint $\pi_{HH}>U_{0}$ is automatically satisfied, i.e., the constraint (26) is a redundant constraint.

### Appendix A.2. Proof for Proposition 2

**Proof** **1.** The information rent of the efficient manufacturer of carbon reduction can be stated as

$$\Delta =\pi_{H}-\pi_{m}=\frac{k_{H}{\left(\alpha -w\beta \right)}^{2}}{4k_{H}\beta -2\gamma^{2}}-\frac{k_{L}{\left(\alpha -w\beta \right)}^{2}}{4k_{L}\beta -2\gamma^{2}}=\frac{\gamma^{2}{\left(\alpha -w\beta \right)}^{2}\left(k_{L}-k_{H}\right)}{2\left(2k_{H}\beta -\gamma^{2}\right)\left(2k_{L}\beta -\gamma^{2}\right)}=\frac{\gamma^{2}{\left(\alpha -w\beta \right)}^{2}\left(k_{L}-k_{H}\right)}{2\left(2k_{H}\beta -\gamma^{2}\right)\left(2\left(k_{H}+\left(k_{L}-k_{H}\right)\right)\beta -\gamma^{2}\right)}$$

It is easy to obtain $\partial \Delta /\partial (k_{H}-k_{L})<0$, thus we get the proposition. □

### Appendix A.3. Proof for Proposition 4

**Proof** **2.** When Equation (24) is satisfied, substitute Equations (27) and (28) into Equation (24). Thus, we have

$$\frac{k_{H}\beta [\alpha -\beta (w_{H}+w_{L})](w_{L}-w_{H})}{4k_{H}\beta -2\gamma^{2}}\geq T_{H}-T_{L}$$

Accordingly

$$\begin{array}{cc} \pi_{LL}-\pi_{LH} & =\frac{k_{L}\beta \left[2\alpha -\beta \left(w_{H}+w_{L}\right)\right]\left(w_{H}-w_{L}\right)}{4k_{L}\beta -2\gamma^{2}}-T_{L}+T_{H}\\ & \geq \frac{k_{L}\beta \left[2\alpha -\beta \left(w_{H}+w_{L}\right)\right]\left(w_{H}-w_{L}\right)}{4k_{L}\beta -2\gamma^{2}}+\frac{k_{H}\beta \left[\alpha -\beta \left(w_{H}+w_{L}\right)\right]\left(w_{H}-w_{L}\right)}{4k_{H}\beta -2\gamma^{2}}\\ & =\frac{2\beta \gamma^{2}\left[2\alpha -\beta \left(w_{H}+w_{L}\right)\right]\left(w_{H}-w_{L}\right)\left(k_{L}-k_{H}\right)}{\left(4k_{L}\beta -2\gamma^{2}\right)\left(4k_{H}\beta -2\gamma^{2}\right)}>0 \end{array}$$

That is $\pi_{LL}\geq \pi_{LH}$, i.e., the constraint (23) is redundant. □
